# Upper Airway Resistance Syndrome Patients Have Worse Sleep Quality Compared to Mild Obstructive Sleep Apnea

**DOI:** 10.1371/journal.pone.0156244

**Published:** 2016-05-26

**Authors:** Luciana Balester Mello de Godoy, Gabriela Pontes Luz, Luciana Oliveira Palombini, Luciana Oliveira e Silva, Wilson Hoshino, Thaís Moura Guimarães, Sergio Tufik, Lia Bittencourt, Sonia Maria Togeiro

**Affiliations:** Universidade Federal de São Paulo, Departamento de Psicobiologia, São Paulo, Brasil; Charité - Universitätsmedizin Berlin, GERMANY

## Abstract

**Purpose:**

To compare sleep quality and sustained attention of patients with Upper Airway Resistance Syndrome (UARS), mild Obstructive Sleep Apnea (OSA) and normal individuals.

**Methods:**

UARS criteria were presence of excessive daytime sleepiness (Epworth Sleepiness Scale—ESS—≥ 10) and/or fatigue (Modified Fatigue Impact Scale—MFIS—≥ 38) associated to Apnea/hypopnea index (AHI) ≤ 5 and Respiratory Disturbance Index (RDI) > 5 events/hour of sleep or more than 30% of total sleep time with flow limitation. Mild OSA was considered if the presence of excessive daytime sleepiness (ESS ≥ 10) and/or fatigue (MFIS ≥ 38) associated to AHI ≥ 5 and ≤ 15 events/hour. “Control group” criteria were AHI < 5 events/hour and RDI ≤ 5 events/hour and ESS ≤ 9, without any sleep, clinical, neurological or psychiatric disorder. 115 individuals (34 UARS and 47 mild OSA patients and 34 individuals in “control group”), adjusted for age, gender, body mass index (BMI) and schooling years, performed sleep questionnaires and sustained attention evaluation. Psychomotor Vigilance Task (PVT) was performed five times (each two hours) from 8 a.m. to 4 p.m.

**Results:**

UARS patients had worse sleep quality (Functional Outcomes of Sleep Questionnaire—FOSQ—and Pittsburgh Sleep Quality Index—PSQI: p < 0.05) and more fatigue than mild OSA patients (p = 0.003) and scored significantly higher in both Beck inventories than “control group” (p < 0.02). UARS patients had more lapses early in the morning (in time 1) compared to the results in the afternoon (time 5) than mild OSA (p = 0.02). Mild OSA patients had more lapses in times 2 than in time 5 compared to “control group” (p = 0.04).

**Conclusions:**

UARS patients have a worse sleep quality, more fatigue and a worse early morning sustained attention compared to mild OSA. These last had a worse sustained attention than controls.

## Introduction

Sleep related breathing disorders (SRBD) is a frequent sleep disorder associated with significant clinical consequences. The physiopathology, consequences and treatment of moderate to severe Obstructice Sleep Apnea (OSA) had already been thoroughly researched for several authors. However, these aspects are still controversial on mild OSA. Recently, one study showed that patients with mild OSA presented more lapses during sustained attention evaluation compared to normal subjects [1–3].

Mild SRBD includes mild OSA and Upper Airway Resistance Syndrome (UARS). UARS was first described in 1993 as a sleep breathing disorder characterized by a complaint of excessive daytime sleepiness (EDS) and frequent awakenings during sleep due to increased respiratory effort [4]. According to the review of Exar and Collop (1999), the most suitable cause of UARS daytime sleepiness is the sleep disruption due to increased number of brief electroencephalographic (EEG) arousals associated to increase in negative intrathoracic pressure and inspiratory flow limitation events [5]. After their study, several authors evaluated the clinical features of this syndrome [6–10] and tried to define better its characteristics. However, whether UARS is a distinct disease or the onset of OSA is still controversial.

Stoohs and colleagues (2008) showed that patients with UARS report higher incidence of chronic insomnia, EDS and fatigue than patients with OSA, however, OSA patients present more severe upper airway abnormalities leading to pharyngeal collapse, compared to UARS patients [10]. The less severe upper airway collapsibility in UARS may be explained by their frequent arousal and sleep fragmentation [11]. It has been suggested that sleep disruption “protects” from the consequent oxyhemoglobin desaturation that occurs after more severe obstructive events such as apnea and hypopnea as in OSA [11].

By analyzing OSA and UARS polysomnographic data, Multiple Sleep Latency Test (MSLT) and spectral analysis of the EEG during sleep, it was observed that individuals with UARS have a higher amount of alpha-frequency central lead EEG compared to patients OSA. The authors suggest that patients with UARS and OSA have differences in cortical response to respiratory changes during sleep. The increased number of brief EEG arousals after inspiratory flow limitation events indicate an attempt to prevent upper airway (UA) collapse. This mechanism of UA protection is preserved in UARS patients, but OSA patients present significant impairment [12].

It is still not well established the clinical consequences of UARS, and it is not well defined also the different aspects of UARS compared to mild OSA patients. Therefore, the objective of this study was to compare the sleep quality, mood and sustained attention of UARS patients to mild OSA.

## Methods

This project was approved by the Research and Ethics Committee (N° 1300/11) of the Federal University of São Paulo, and all volunteers signed an informed consent form before data collection. It was registered in Clinical Trials as NCT01461486. Individuals of either gender, between the ages of 25 and 50 years, with a body mass index (BMI) ≤ 35 kg/m^2^ were included. The method of sampling was not-probabilistic consecutive. These patients were recruited from Outpatient Clinic of Sleep Breathing Disorders at Universidade Federal de São Paulo (2011–2014). Patients with UARS were selected according to polysomnographic features as Respiratory Disturbance Index (RDI) score ≥ 5 events/hour or time in inspiratory flow limitation > 30% of total sleep time and Apnea- hypopnea Index (AHI) < 5 events/hour and associated to clinical criteria of fatigue (Modified Fatigue Impact Scale—MFIS—≥ 38) [13] and/or excessive daytime sleepiness (Epworth Sleepiness Scale—ESS ≥ 10) [14]. Mild OSA was considered if patients had AHI ≥ 5 and ≤ 15 events/hour of sleep [15, 16] and had fatigue (Modified Fatigue Impact Scale—MFIS—≥ 38) [13] and/or excessive daytime sleepiness (Epworth Sleepiness Scale—ESS ≥ 10) [14]. The “control group” consisted of individuals with AHI < 5 events/hour and RDI ≤ 5 events/hour, ESS ≤ 9 and without any sleep, clinical, neurological or psychiatric disorder.

Patients with a predominance of central apnea during polysomnography (PSG); abusive use of alcohol and psychoactive drugs; a decompensated clinical disease, or neurological or psychiatric disorder; other sleep disorders (insomnia, restless legs syndrome, bruxism, narcolepsy, parasomnias, and sleep—wake circadian disorders) and those who had already received previous sleep treatments or were unable to fully participate in the study were excluded. These exclusion criteria were checked by a physician during medical evaluation.

All groups performed a full-night PSG. The exam was performed in the sleep laboratory of the Research Incentive Fund Association (Associação Fundo de Incentivo à Pesquisa, AFIP), Sleep Institute using a digital PSG system (Embla^®^S7000, Embla Systems, Inc., Broomfield, CO, USA).

A full-night attended PSG was performed in each subject and sleep stages, arousals, and leg movements were scored according to the standard criteria [17]. Apneas and hypopneas were scored following the recommended AASM rules [17]. Hypopneas were scored by the AASM “alternative” rules, i.e., when a > 50% reduction in airflow amplitude was observed on the nasal cannula signal lasting ≥ 10 seconds and accompanied by a decrease of ≥ 3% in oxyhemoglobin saturation (SpO2) or an arousal.

Inspiratory flow limitation (IFL) was scored manually and visually identified as a “flattened shape” of the inspiratory airflow contour at nasal cannula pressure from Embla system (square root of the flow signal), with no filters applied. At least four consecutive breaths with “flattened shape” were required to score IFL events [18, 19]. Those events should not meet the criteria for hypopnea. The percent of total sleep time (TST) during which there was IFL was calculated.

Following the night that the PSG was performed, the volunteers in both groups remained in the sleep laboratory during the day for the collection of baseline data.

The volunteers completed the following questionnaires: the MFIS [13]; Pittsburgh Sleep Quality Index (PSQI) [20, 21]; ESS [14, 22]; Beck Anxiety Inventory (BAI) [23]; Beck Depression Inventory (BDI) [23] and Functional Outcomes of Sleep Questionnaire (FOSQ) [24]. The volunteers also performed five Psychomotor Vigilance Task (PVT) [25] measurements 5 times each 2 hours over the course of the day.

The sustained attention test was carried out using a portable PVT monitor (model PVT-192, CWE, Inc., Ardmore, PA, USA) to evaluate the state of alertness, psychomotor performance, sustained attention and vigilance over a 10-min period. The task consists of responding to a small, bright red-light stimulus (LED digital counter) by pressing a button as soon as it appears. The stimuli are consecutive and random and occur over the range of 2 to 10 s per minute. The participants were instructed to press a button as soon as each stimulus appears, but not too soon, as this yielded a false start warning on the display [23].

Statistical analysis was performed using the SPSS statistics software (version 21.0 for Windows). For characterization of the groups, we performed a descriptive analysis mean ± standard deviation, β coefficient, and confidence interval, considering α ≤ 0.05.

Descriptive variables were analyzed through Univariate General Linear Model (GLM). The test Generalized Linear Model (GLzMM) was used with the following variables of interest: sleepiness, fatigue, sleep quality, quality of life, subjective clues of anxiety and depression and polysomnographic variables, adjusting for age, BMI and schooling years. The choice of distribution considered was based on parsimony between the exploratory analysis of histograms and in the balance good fit Akaike information criterion (AIC) and Bayesian information criterion (BIC). Thus, the variables of these questionnaires were evaluated by Poisson distributions and the variables of PSG were analyzed by Tweedie distribution. For analysis of the PVT repeated-measures, the test Generalized Estimating Equation with Gamma distribution was used.

## Results

Initially, 207 volunteers were selected for the study, although 141 of these subjects did not meet the inclusion criteria and were excluded at the beginning of the project. As a result, 47 individuals with mild OSA and 34 with UARS were included. The participants of both groups were recruited by the Sleep Disorders Ambulatory Clinic of AFIP from December 2011 to August 2014 and then referred to the research project. The participants in the “control group” were recruited by relatives of patients and the media and consisted of 34 individuals.

We had adjusted these 115 individuals for age, BMI and schooling years (Table 1). From these 115 individuals, 50 (3,5%) were men and 65 (56,5%) were women. Mean age was 42.68 ± 9.74 years old. Mean BMI was 26.46 ± 3.84 kg/m^2^ and mean neck circumference was 37.59 ± 3.71 cm. Mean schooling years were 14.43 ± 3.92 years.

**Table 1:** Descriptive data.

Descriptive variables	Control (n = 34)	UARS (n = 34)	Mild OSA (n = 47)	F	Effect size	Observed power
Gender (M/F)	15/19	14/20	21/26	-	-	-
Age (years)	36.76±8.99	42.91±8.0*	46.67±9.37**	12.16	0.18	0.99
BMI (kg/m^2^)	24.40±3.12	25.94±3.94	28.28±3.41**^£^	12.43	0.18	0.99
Schooling years	16.20±4.16	13.76±3.93*	13.59±3.31**	5.42	0.09	0.84

* p < 0.05 between control group and UARS

** p < 0.05 between control group and Mild OSA

^£^ p < 0.05 UARS group and Mild OSA

Data represented by Mean ± Standard deviation and 95% Confidence Interval (lower and upper); General Linear Model (GLM)

Patients with UARS and mild OSA were significantly more sleepy than individuals of the “control group” (p < 0.001) according to the ESS, as expected by inclusion criteria and had also more fatigue according to the MFIS (p < 0.001). Patients with UARS also had significantly more fatigue complaints than those with mild OSA (p = 0.003) (Table 2). UARS patients had a greater score in Pittsburgh Sleep Quality Index (PSQI) component 1 (daytime dysfunction) than the “control group” (p = 0.02). UARS and mild OSA patients had greater scores in PSQI component 6 (subjective sleep quality) than “control group”(p < 0.001 and p = 0.03 respectively). Patients with UARS had also greater score in PSQI component 6 than mild OSA (p < 0.001). Mild OSA patients had greater score in PSQI component 7 than “control group” (p = 0.03). There were no significant differences when we compared the three groups’ results of the components 2, 3, 4, 5. UARS patients had greater score in the global score of PSQI in comparison to “control group” and to mild OSA patients (p < 0.001 and p = 0.02 respectively). Mild OSA patients had greater scores in PSQI global score than “control group’ (p = 0.003) (Table 2).

**Table 2:** Questionnaires.

Questionnaires	Control (n = 34)		UARS (n = 34)		Mild OSA (n = 47)	
	Mean ± SD	95% CI	Mean ± SD	95% CI	Mean ± SD	95% CI
Epworth	6.43±2.9	5.39–7.48	12.70 ±4.37*	11.10–14.31	12.45±4.82**	11.02–13.89
Fatigue	27.83±16.23	21.42–33.13	47.38±17.42*	40.99–53.77	44.15±16.88**^£^	39.13–49.16
Pittsburg 1	1.21±0.65	0.98–1.45	1.93±1.06*	1.54–2.32	1.73±0.61	1.55–1.92
Pittsburg 2	0.90±0.77	0.62–1.18	1.35±1.14	0.93–1.77	1.26±0.95	0.97–1.54
Pittsburg 3	1.25±0.80	0.96–1.53	1.54±0.92	1.20–1.88	1.47±0.88	1.21–1.74
Pittsburg 4	0.68±0.99	0.32–1.04	0.74±0.96	0.38–1.09	1.00±1.13	0.66–1.33
Pittsburg 5	1.18±0.69	0.93–1.43	1.83±0.68	1.58–2.09	1.78±0.59	1.60–1.95
Pittsburg 6	0.12±0.42	(-0.02)-0.27	1.77±0.76*	1.49–2.05	0.36±0.92**^£^	0.09–0.64
Pittsburg 7	0.75±0.84	0.44–1.05	1.09±1.01	0.72–1.46	1.47±0.80**	1.23–1.71
Pittsburg Total	6.12±2.99	5.04–7.20	10.61±4.81*	8.84–12.37	9.10±3.02**^£^	8.21–10.00
FOSQ General productivity	3.52±0.45	3.35–3.68	2.83±0.81*	2.53–3.13	3.22±0.55^£^	3.05–3.39
FOSQ Social outcome	3.70±0.48	3.35–4.12	2.95±1.01*	2.58–3.32	3.32±0.78^£^	3.09–3.55
FOSQ Activity level	3.35±0.46	3.18–3.51	2.72±0.85*	2.42–3.02	3.01±0.59^£^	2.84–3.18
FOSQ Vigilance	3.46±0.53	3.27–3.65	2.71±0.85*	2.39–3.02	3.03±0.66^£^	2.83–3.22
FOSQ Intimate Relationshipsd and Sexual Activity	3.34±0.81	3.04–3.63	2.71±1.21	2.27–3.16	3.17±0.86^£^	2.92–3.43
FOSQ Total	17.28±2.29	16.46–18.11	13.50±3.65*	12.16–14.84	15.42±3.08^£^	14.50–16.33
BECK Anxiety	7.65±8.50	4.58–10.72	19.22±14.41*	13.93–24.51	13.82±11.88^£^	10.29–17.35
BECK Depression	8.18±5.69	6.13–10.24	17.48±10.84*	13.50–21.46	13.10±6.96**^£^	11.03–15.17

* p < 0.05 between control group and UARS

** p < 0.05 between control group and Mild OSA

^£^ p < 0.05 UARS group and Mild OSA

Data represented by Mean ± Standard deviation and 95% Confidence Interval (lower and upper); Generalized Linear Model (GLzMM)

According to FOSQ, UARS patients had a worse general productivity, social outcome, activity level, vigilance and total score than “control group” (p = 0.001, p = 0.003, p < 0.001, p < 0.001, p < 0.001, respectively) and than mild OSA patients (p = 0.001, p = 0.045, p = 0.02, p = 0.03, p = 0.002, respectively). Also, UARS patients had a worse FOSQ intimate relationships and sexual activity scores than mild OSA patients (both p = 0.04) (Table 2).

When mood was analyzed by Beck inventories, UARS patients scored significantly higher in Anxiety (BAI) and Depression (BDI) inventories compared to “control group” (p = 0.001 and p < 0.001, respectively) and to mild OSA patients (p = 0.008 and p = 0.02, respectively). Mild OSA patients scored significantly higher in BDI than “control group” (p = 0.04) (Table 2).

As expected, there were some statistically significant differences when comparing the polysomnographic variables of the three groups (Table 3).

**Table 3:** Polysomnographic variables.

PSG variables	Control (n = 25)		UARS (n = 25)		Mild OSA (n = 25)	
	Mean ± SD	95% CI	Mean ± SD	95% CI	Mean ± SD	95% CI
Sleep latency	11.40±2.07	7.98–16.30	18.25±2.95*	13.28–25.07	22.06±3.41	16.29–29.87
REM sleep latency	116.48±11.43	96.08–143.56	94.54±9.78	77.18–115.79	102.66±10.40	84.16–125.21
Total sleep time (min)	384.84±12.97	360.24–411.13	355.41±12.22	332.25–380.19	370.55±12.61	346.64–396.11
Sleep eficiency (%)	87.99±2.27	83.63–92.57	81.66±2.15	77.54–85.99	82.63±2.17	78.48–87.00
N 1 (%)	8.58±0.83	7.08–10.39	11.21±1.02	9.37–13.41	11.53±1.04	9.65–13.78
N 2 (%)	46.32±1.55	43.37–49.47	48.13±1.60	45.09–51.37	45.94±1.54	43.04–49.08
N 3(%)	24.30±1.41	21.68–27.24	21.57±1.29	19.17–24.26	21.19±1.27	18.82–23.85
REM (%)	20.76±1.34	18.28–23.58	19.09±1.26	16.76–21.73	21.31±1.37	18.78–24.18
Arousal index	8.90±0.76	7.52–10.52	15.24±1.13*	13.16–17.64	16.24±1.19**	14.05–18.76
RDI	3.13±0.47	2.32–4.21	15.04±1.54*	12.31–18.39	14.40±1.49**	11.75–17.64
AHI	1.62±0.20	1.16–2.25	4.11±0.54*	3.16–5.33	8.86±0.97**^£^	7.15–10.99
Mean SpO_2_	95.63±0.32	95.00–96.27	95.75±0.32	95.12–96.39	95.08±0.32	95.12–96.39
Minimum SpO_2_	86.40±3.31	80.13–93.15	89.08±3.39	82.66–95.99	85.64±3.29	79.41–92.35
REM desaturation index	3.92±0.93	2.45–6.26	6.07±1.30	3.98–9.24	17.98±2.94**^£^	13.05–24.78
NREM desaturation index	1.03±0.23	0.66–1.60	2.07±0.39*	1.43–2.99	7.45±1.01**^£^	5.70–9.74
Desaturation < 90%	7.90±2.05	4.74–13.15	0.11±0.08*	0.02–0.49	2.76±0.93^£^	1.43–5.36

* p < 0.05 between control group and UARS

** p < 0.05 between control group and Mild OSA

^£^ p < 0.05 between UARS group and Mild OSA

Data represented by Mean ± Standard deviation and 95% Confidence Interval (lower and upper); Generalized Linear Model (GLzMM)

When sustained attention was analyzed with PVT, we observed that the mean reaction time in time 3 was greater than in time 5, independently of the group (p = 0,04) (Table 4).

**Table 4:** Psychomotor Vigilance Test (PVT) variables.

PVT	Control (n = 34)		UARS (n = 34)		Mild OSA (n = 47)	
	Mean ± SD	95% CI	Mean ± SD	95% CI	Mean ± SD	95% CI
Time	***Mean RT***					
1	298.57±320.94	265.66–320.83	314.20±337.30	286.31–341.48	295.38±316.25	276.16–323.09
2	291.26±311.24	258.995–313.54	282.94±303.75	255.08–309.62	296.30±316.68	276.70–323.09
3‡	292.11±311.82	253.853–320.13	277.89±298.32	244.52–310.80	304.82±328.35	281.12–337.49
4	293.76±314.62	261.531–316.01	281.90±302.40	254.69–309.17	287.58±308.96	268.83–315.17
5	289.60±310.78	259.097–309.80	275.35±295.73	240.91–291.61	281.16±300.32	263.74–306.86
Total average	287.80±13.50	265.658–320.83	284.42±9.66	286.31–341.48	297.21±12.88	276.16–323.09
Time	***False Start***					
1	1.37±1.05	-0.50–3.23	2.29±0.97	0.50–4.23	2.61±0.89	1.07–4.23
2	2.18±1.77	-0.79–5.52	3.59±1.63	0.42–6.73	4.91±1.49	2.29–7.63
3	1.85±2.25	-2.11–5.87	5.01±2.07	1.25–9.23	5.07±1.90	1.45–8.21
4	0.97±2,36	-2.66–5.75	4.95±2.18	1.01–9.42	6.25±1.99	2.20–9.32
5	2.24±2.21	-1.75–6.11	4.89±2.07	1.28–9.14	5.39±1.86	1.89–8.55
Total average	1.86±0.42	0.99–2.73	4.32±1.19	1.89–6.74	4.68±1.97	0.70–8.66
Time	***Lapses***					
1	3.34±11.55	2.34–4.36	4.20±14.56	3.10–5.17	3.08±10.60£	2.34–4.06
2	2.89±9.94	1.91–3.97	2.81±9.73	1.68–3.74	3.34±11.49**	2.47–4.23
3	3.06±10.54	2.09–4.10	3.00±10.41	1.95–3.96	3.14±10.81	2.36–4.07
4	3.30±11.40	2.38–4.26	3.03±10.48	2.05–3.93	2.96±10.22	2.25–3.86
5	3.16±10.91	2.27–4.18	2.52±8.74	1.19–3.09	2.75±9.46	2.01–3.63
Total average	3.20±0.56	2.06–4.34	3.04±0.33	2.36–3.73	3.13±0.40	2.32–3.94
Time	***Total Errors***					
1‡	1.50±1.08	-0.43–3.40	2.45±0.99	0.60–4.43	2.65±0.91	1.08–4.31
2	2.36±1.81	-0.66–5.81	3.77±1.67	0.52–6.99	5.07±1.53	2.37–7.85
3	2.05±2.32	-2.02–6.20	5.25±2.13	1.41–9.62	5.21±1.96	1.45–8.41
4	1.10±2.40	-2.58–5.97	5.31±2.21	1.30–9.85	6.42±2.03	2.29–9.53
5	2.41±2.29	-1.73–6.45	5.15±2.11	1.42–9.61	5.76±1.94	2.08–9.01
Total average	20.4±0.46	1.10–2.98	4.57±1.26	1.99–7.16	4.83±2.00	0.80–8.87
Time	***Mean RT < 100ms***					
1	0.97±2.76	-0.03–0.09	1.02±2.84	-0.004–0.12	0.97±2.74	-0.03–0.08
2	1.36±3.95	-0.01–0.37	1.69±4.53	0.05–0.43	1.50±4.21	-0.07–0.25
3	0.98±2.81	0.01–0.29	1.33±3.61	0.07–0.35	1.03±2.88	-0.05–0.19
4	0.94±2.71	-0.07–0.13	1.29±3.66	0.05–0.26	1.01±2.81	-0.02–0.15
5	1.47±4.24	-0.05–0.24	1.33±3.81	0.10–0.39	1.01±2.87	-0.01–0.23
Total average	1.12±3.23	0.004–315.73	1.32±3.64	0.006–300.36	1.09±3.06	0.004–267.62
Time	***Mean fastest RT***					
1	213.26±148.04	198.76–223.66	210.98±148.32	198.68–223.59	210.91±147.30	201.58–222.76
2	208.91±143.99	194.03–220.00	199.20±139.92	186.27–212.24	208.40±145.24	198.37–220.46
3	209.54±143.62	193.69–221.41	198.41±139.66	184.68–212.40	204.61±142.96	194.28–217.85
4	212.18±146.14	198.02–222.61	199.10±140.09	186.97–211.56	198.71–139.54	189.67–210.59
5	210.95±145.15	194.35–223.93	194.51±136.82	175.72–205.31	202.90±141.85	191.42–216.58
Total average	287.81±13.50	260.30–315.31	284.41±9.65	264.77–304.06	297.20±12.88	271.26–323.15

** p < 0.05 between this time and time 5 ("control group" and mild OSA)

^£^ p < 0.05 between this time and time 5 (UARS and mild OSA)

^‡^ p < 0.05 between this time and time 5 independently of the group

Reference: time 5 and group mild OSA; General Estimation Equation Test (time, group, interaction time x group)

**Fig 1:**
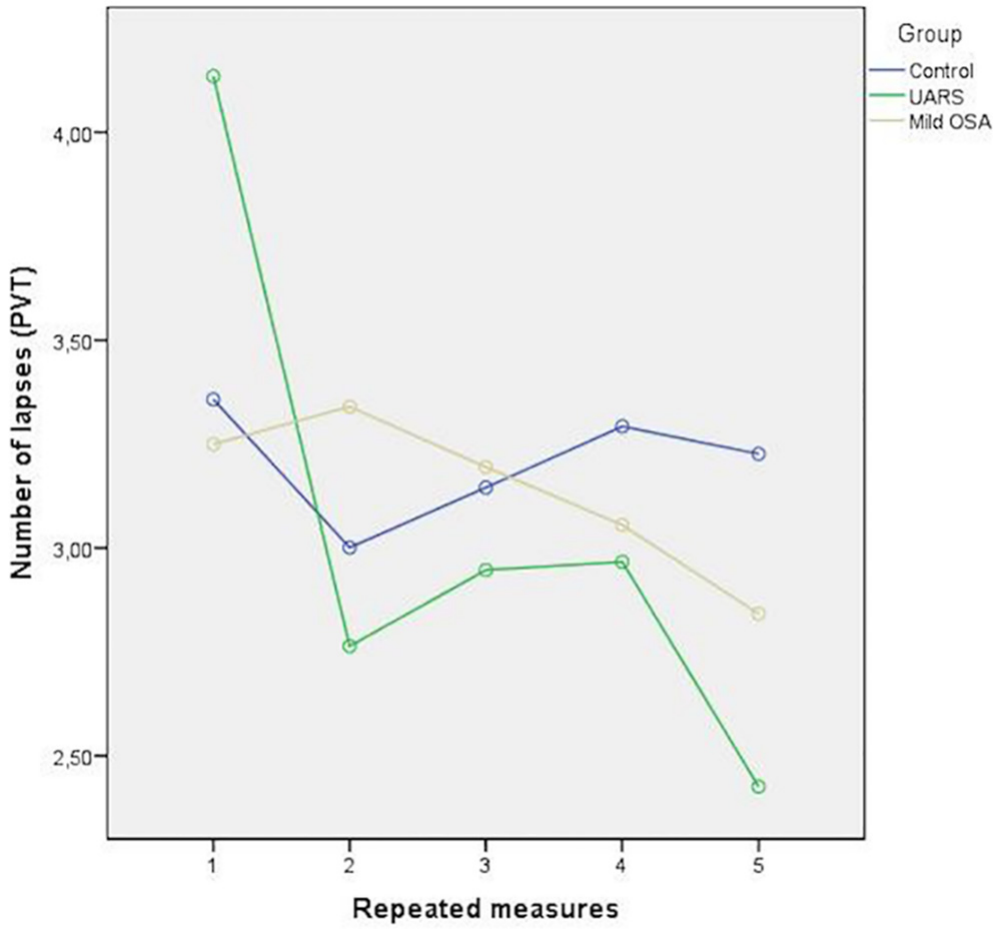
Number of lapses of PVT repeated measures: mild OSA patients had more lapses at times 2 than at time 5 than “control group” (p = 0.044). UARS patients had more lapses at time 1 than at time 5 in comparison to mild OSA patients (p = 0.02).

Mild OSA patients had more lapses at times 2 than at time 5 than “control group” (p = 0.044). UARS patients had more lapses at time 1 than at time 5 in comparison to mild OSA patients (p = 0.02) (Fig 1 and Table 4).

There was not any statistical significant difference in false starts, total errors, number of reaction time less than 100ms and the fastest 10% of response times’ results (Table 4).

## Discussion

In our study, we demonstrated that mild OSA patients had a worse subjective sleep quality and had higher score in depression inventory than “control group”. Mild OSA patients had also more lapses in the morning than in the afternoon compared to “control group”. UARS patients had worse daytime dysfunction, subjective sleep quality, general productivity, social outcome, activity level, vigilance and had higher score in anxiety and depression inventoties than “control group”. UARS and mild OSAS patients also demonstrated significant differences in clinical complaints. Therefore, we can say that mild SRBD have a significant clinical impact.

Currently there are controversies about the associations of cardiovascular diseases and mild SRBD [26–28], as well as, regarding clinical symptoms of this group of patients. Some authors evaluated mild OSA patients’ quality of life, subjective sleepiness and mood and compared their results with healthy individuals and did not find statistically significant difference [29, 30]. Otherwise, some authors found cognitive deficit (PVT) in patients with mild OSA [31]. Luz and colleagues (2015), for instance, compared PVT of mild OSA patients with normal individuals and found that those patients had more lapses than normal individuals, in accordance with our results [1].

In our study, UARS defined as the presence of excessive daytime sleepiness and/or fatigue associated to AHI ≤ 5 and RDI > 5 events/hour of sleep or more than 30% of total sleep time with flow limitation was associated with more clinical complaints compared to mild OSA. UARS patients had more fatigue, worse subjective sleep quality, impaired general productivity, social outcome, activity level, vigilance, intimate relationships and sexual activity and had higher scores in anxiety and depression inventories than mild OSA patients. UARS patients also had more lapses early in the morning than mild OSA.

UARS has been described for more than 15 years. However, there is still no consensus on the diagnostic criteria and whether UARS represents a distinct syndrome from OSA. The results of this study demonstrated that UARS patients defined by specific criteria present significantly worse subjective sleep quality, impaired daytime quality of life, more tendencies to mood disorders and decrease in early morning attention compared to mild OSA patients.

The respiratory parameters in polysomnography for mild SRBD have been subject of controversies and different aspects do not have a consensus yet. Different diagnostic criteria have been used for UARS. RERAs have been recognized as events associated with increased upper airway resistance [9, 11, 19]. Even though the latest ICSD-3 criteria include the presence of 5 or more obstructive events, including apnea, hypopnea and RERAs, the cut-off of 5 events /hour, it is not established yet as a diagnostic consensus and follow-up studies evaluating significant clinical outcomes are necessary to confirm these criteria. The % of total sleep time with flow limitation has also been suggested as a respiratory criterion to identify SRBD. However it is not defined yet what cutoff value should be used. 30% of total sleep time with flow limitation was identified in a group of normal individuals selected from a general population study [18]. Based on this study we used more than 30% criterion for flow limitation to characterize UARS patients. However, this criterion is not validated yet.

Excessive daytime sleepiness and fatigue are the most disabling symptoms of SRBD. It is associated with worse quality of life, higher risk of motor vehicle accidents and work-related incidents. The most common complaints of UARS patients are EDS and fatigue, despite an adequate number of hours of sleep. Most patients claim of non-restorative sleep, tiredness and difficulty in performing daily activities. In our study UARS patients presented significantly more complaints of fatigue than mild OSA. Previous studies already demonstrated that UARS patients present more complaints than OSA patients [3, 10, 11], but it is still not well defined the clinical differences between UARS and mild OSA patients.

Sleep fragmentation might be the main cause of EDS in UARS and is due to an increased number of brief EEG arousals after inspiratory flow limitation events. These events are associated to abnormalities in sleep structure which may lead to impaired daily functioning. However, the consequences of UARS in sleep structure may be underrecognized since conventional parameters may not reflect the sleep abnormalities in this SRBD. Other parameters that may improve the recognition of these abnormalities are Cyclic Alternating Pattern (CAP) analysis and spectral analysis of EEG.

UARS patients may present consequences in sleep structure that are not identified by conventional polysomnography parameters. Black and colleagues (2000) demonstrated more subtle electroencephalographic changes after the inspiratory effort that were not visually discernible [12]. They performed a spectral analysis throuh Fast Fourier Transformation (FFTs) and found that esophageal pressure (Pes) reversal was preceded by increases in delta activation regardless the presence or not of arousals. According to the authors, these events may reflect changes in brain function as a consequence of the respiratory event. We did not find any difference comparing sleep architecture conventional parameters of UARS and mild OSA in our study; however, we did not perform a spectral analysis of the EEG of these patients.

These subtle abnormalities in sleep architecture in UARS patients might explain their worse sleep quality and worse complaints regarding daily functioning compared to mild OSAS. In some aspects, UARS is considered an extension of the spectrum of SRBD associated with milder degrees of pharyngeal collapse and consequent changes in sleep structure. Indeed, UARS patients have inspiratory flow limitation during sleep. It is not defined yet how much of flow limitation is sufficient to account for the clinical characteristics of patients with UARS [9]. Gold and colleagues (2008) tried to determine whether the severity of hypersomnolence observed in UARS patients could be predicted from the patterns observed among OSA patients [9]. They observed that the severity of hypersomnolence decreased over the continuum from severe to mild OSA. Nevertheless, the model significantly underestimated hypersomnolence in UARS patients. UARS patients were sleepier than the model predicted and also had higher frequency of sleep-onset insomnia. Some authors had also suggested that UARS symptoms closely resemble those of the functional somatic syndromes, in addition to excessive daytime sleepiness and sleep-onset insomnia, including symptoms such as unrefreshing sleep, sleep fragmentation, bruxism, muscle pain, heartburn, diarrhea, headaches, depression and orthostatic syncope [8]. These findings suggest that UARS patients might have a worse sleep quality and consequent more impaired daily functioning than mild OSA patients.

The difference in clinical complaints between UARS and mild OSA patients may also be influenced by different personality characteristics [32, 33]. So and colleagues (2015) compared UARS patients (AHI < 10 events/hour with flow limitation and RERA index ≥ AHI with ESS ≥ 7) with OSA patients (AHI ≥ 10 events/hour) and found that UARS patients tend to have more neurotic, anxious and sensitive personalities than OSA patients [3].

Excessive daytime sleepiness is associated with decrements in quality of life and impaired sustained attention [34, 35]. Decreased reaction time (RT) caused by SRBD daytime sleepiness has been implicated in increased motor vehicle accidents [36] and is reversible after OSA treatment [37]. PVT is a sustained-attention, reaction-timed task that measures the speed by which subjects respond to a visual stimulus. In our study, we performed the test 5 times each 2 hour during the day and analyzed the RT, number of lapses, errors and false starts, number of RT < 100ms and the 10% fastest RT. Both OSA and UARS can cause cognitive changes and excessive daytime sleepiness that decrease RT to time-sensitive tasks [38]. Nevertheless, some authors suggested that UARS patients might present a central nervous system hyper reactivity during sleep since they found a significantly higher power density in the slow a-EEG (7–9 Hz) and the low D band (0.5–2 Hz) in UARS patients compared to OSA patients [39]. This may be at least one of the explanations for our results demonstrating that UARS patients had more lapses early in the morning compared to the results in the afternoon than mild OSA patients. Stoohs and colleagues (2009) compared PVT results performed in UARS patients (mean AHI = 3.5 ± 1.4 events/hour) with OSA patients (mean AHI = 17.5 ±12.2 events/hour) and also found worse results in UARS group on most PVT measures except the number of errors [40]. Moreover, unlikely our study with only mild OSA patients, they also included patients with moderate OSA in their data.

This is pioneering study that intended to better describe the different features of the mild SRBD. By using the similar symptomatic criteria (EDS and fatigue) of mild OSA and UARS and adjusting the analysis by age, gender, BMI and schooling years, we decreased the possible selection bias. On the other hand, this study has some limitations. Most of our analysis was based on subjetive questionnaires. The only objective test performed was PVT. Objetive evaluation of sleep quality and EDS, for example, would be important to better define the characteristics of the different mild SRBD. Another limitation is the UARS definition used in this study, which is not validated yet. Currently the diagnostic criteria for UARS is still not well defined. Many authors believe that it is part of OSA spectrum. However, some distinct clinical characteristics have been described in UARS patients. The different features between UARS and OSA need to be better investigated and established, since it will improve patient identification and more efficient treatment indication.

In summary, mild SRBD patients were more sleepy, fatigue, anxious, depressive and had more lapses during sustained attention test compared to normal control. On the other hand, UARS patients presented significant more clinical complaints related to daytime function, more fatigue, worse sleep quality, impared mood and worse early morning sustained attention compared to mild OSA patients. The results of this study are in agreement with the concept that UARS patients may present different complaints comparing to mild OSA, suggesting that it may represent a distinct sleep disorder. The diagnostic criteria for UARS used in this study are not validated yet, and future studies are necessary to establish the clinical and polysomnographic criteria to identify this SRBD.
